# Immediate implant using an inverted body-shift design versus a conventional conical implant: A randomized clinical trial with 1 year follow-up

**DOI:** 10.4317/medoral.27776

**Published:** 2025-11-22

**Authors:** Andrea Galve-Huertas, Susana García-González, Louis Decadt, Octavi Ortíz-Puigpelat, Federico Hernández-Alfaro, Samir Aboul-Hosn Centenero

**Affiliations:** 1 Department of Oral and Maxilofacial Surgery, Universitat Internacional de Catalunya, Barcelona, Spain

## Abstract

**Background:**

Immediate implant placement is a technique-sensitive procedure, requiring precise positioning on the palatal aspect to ensure ideal prosthetic emergence and contour. Achieving primary stability is essential for the placement of an immediate provisional crown. The Inverted Co-Axis implant is uniquely designed for this purpose, featuring a 12-degree angled neck and a widened middle third, allowing central positioning within the alveolar socket while maintaining primary stability. Given its uncommon use, this study aimed to compare the performance of the Inverted Co-Axis implant with that of a conventional conical implant.

**Material and Methods:**

A randomized controlled trial was conducted with 30 patients, equally divided into two groups (n=15). Immediate implants were placed in the anterior maxilla, each restored with an immediate provisional crown and accompanied by connective tissue grafting during surgery. Outcomes included implant survival, success rates, primary stability (insertion torque, ISQ), bone remodeling, and aesthetic results. Statistical analysis comprised descriptive measures (mean, SD, median, quartiles) and appropriate inferential tests: Mann-Whitney U, Wilcoxon signed-rank, Student's t-test, Chi-square, Kruskal-Wallis, Spearman's correlation, and Brunner-Langer. Significance was set at =0.05.

**Results:**

All implants survived (100% survival rate). Success rates were 93.3% in the control group and 73.3% in the test group, with no statistically significant difference. Insertion torque averaged 35 Ncm (control) and 42 Ncm (test), with ISQ values between 56-62 in both groups. Minimal horizontal bone loss was observed at 1mm, 3mm and 5mm. Vertical bone loss was greater on the buccal aspect in the test group, while palatal loss was higher in the control group. Aesthetic evaluation via the Pink Esthetic Score yielded comparable results.

**Conclusions:**

Both implant designs proved effective and reliable for immediate post-extraction implantation, with favorable outcomes in stability, bone preservation, and aesthetics.

## Introduction

**Figure 1:**
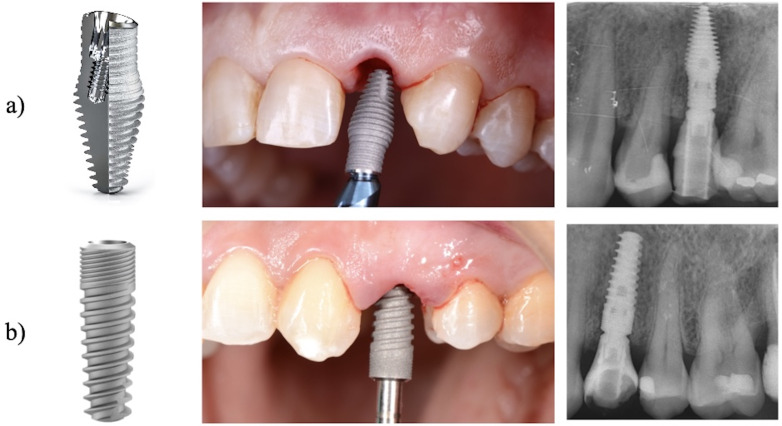
a) Inverted Co-Axis 12º Implant, b) Conventional Conical Implant with internal connection.

In the 1970s and 1980s, implants were traditionally placed only after complete healing, which consequently prolonged treatment. Tooth extraction triggers alveolar bone remodelling, including horizontal and vertical resorption of the buccal and lingual plates. In the anterior region, these changes can compromise aesthetics, particularly if the extraction is not atraumatic (1). In 1978, Wilfred Schulte (University of Tübingen, Germany) introduced the immediate implant concept, placing dental implants directly into extraction sockets at the time of tooth removal. This technique, later refined, aims to shorten treatment time while preserving surrounding bone and soft tissue. Success depends on atraumatic extraction, precise implant positioning for primary stability, and, when appropriate, immediate provisionalization to support peri-implant tissues. The integrity of the extraction socket-particularly the buccal plate-is critical determinant of success, and grafting may be required to manage gaps between the implant and socket walls (1 , 2). Another treatment option for the upper anterior region is early implant placement. In implant dentistry, this refers to placing an implant into the extraction site after a short healing period-typically 4-8 weeks to allow soft tissue healing, or 12-16 weeks for partial bone healing. This timing strikes a balance between the advantages of immediate placement (such as reduced overall treatment time) and delayed placement (which offers more predictable tissue healing). Early implant placement is particularly common in cases involving active periapical infection, where immediate implantation is not advisable (3). Over the past two decades, interest in immediate implant placement has grown considerably. Although technique-sensitive, it has become a routine procedure with success rates comparable to conventional implants. Its advantages include shorter edentulous periods, fewer surgical interventions, minimal invasiveness, better preservation of soft and hard tissues, and improved aesthetic outcomes (4 - 7). For long-term success with immediate implants, several clinical parameters must be carefully observed. Levine et al. (8) outlined ten essential steps for aesthetic-zone cases, including assessing aesthetic risk with the patient and treatment team, using cone beam computed tomography (CBCT) to evaluate bone and root anatomy, performing atraumatic extraction while preserving the buccal plate, placing the implant in an ideal three-dimensional position, selecting a narrower implant to maintain a 2-3mm buccal gap, grafting that gap with a low-resorption biomaterial, augmenting soft tissue when required, immediately shaping the emergence profile with a provisional restoration or customised healing abutment, transferring soft-tissue contours with a custom impression coping, and finally delivering a screw-retained definitive crown rather than using stock abutments or deep cement margins. All of these are considered key steps in the protocol. Because immediate implant placement is highly technique-sensitive, primary stability must be achieved before placing a provisional crown; early provisionalisation assists in managing peri-implant soft tissues, simplifies the restorative phase, and supports an ideal emergence profile (9). Recent preclinical and clinical studies have analysed an innovative macro-hybrid implant design that represents a paradigm shift in biology and aesthetics, addressing limitations that can hinder immediate implants and worsen outcomes (10 - 12). In this design, the implant body is conical in its apical third, widens in the middle third, and then narrows coronally towards the neck (Figure 1a). It can therefore be described as inverted and convergent. At the coronal implant-abutment junction, the profile is narrower where the bone is thinner, more delicate and relatively avascular, allowing a greater volume of graft material to be placed between the implant and the surrounding bone. The middle third widens to enhance primary stability in a region with greater bone volume and vascularisation (13). In addition, a 12° angled neck permits central placement within the socket, optimising alveolar bone use and improving prosthetic emergence; palatal screw access simplifies restoration, avoids cement-retained crowns, and reduces laboratory and prosthetic accessory costs (14 , 15). Despite these promising biomechanical and prosthetic advantages, there is still limited clinical evidence to determine whether this new macro-hybrid design actually translates into superior clinical or radiographic outcomes compared with conventional conical implants in immediate post-extraction sites. Most existing studies are either preclinical or lack randomized clinical comparison. Therefore, additional well-controlled human studies are needed to confirm whether the inverted body-shift design can maintain peri-implant bone levels and soft-tissue stability while improving aesthetic and prosthetic outcomes. Due to the recently introduced design of this 'body-shift' implant, the objective of this study was to compare the inverted co-axis 12º implant with a conventional conical implant commonly used on a daily basis.

## Material and Methods

Study Design and Group This study was a single-blind, randomized controlled clinical trial, conducted at the Universitat Internacional de Catalunya (UIC). Thirty patients were enrolled for the placement of immediate implants with immediate crown placement, to replace maxillary anterior teeth (central and lateral incisors, canines or premolars). The study was registered on ClinicalTrials.gov under ID: NCT06059105. Sample Size Previous studies on this topic identified marginal bone loss as the primary outcome for sample size calculation, since implant survival rates are usually reported as 100% and therefore not useful for detecting group differences. For a reliable estimate, the study by Vandeweghe et al. (11) was used, reporting a mean marginal bone loss of 1.20mm (standard deviation (SD) ±0.215mm) after one year in 15 implants. Based on these data, a two-tailed independent samples t-test (=0.05, power =80%) indicated a required sample size of 30 patients (15 per group). All calculations were performed with G*Power version 3.1.9.7 (Heinrich Heine University, Düsseldorf, Germany). Sample Selection Patients were randomly assigned to two groups. The test group (n=15) received the Inverta co-axis 12° implant (Figure 1a), while the control group (n=15) was treated with a conventional internal conical implant (Figure 1b).

1

**Figure 2:**
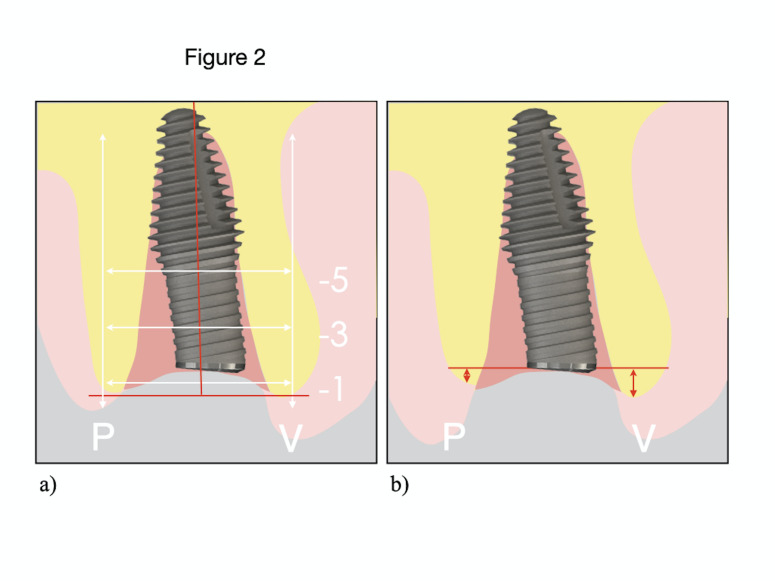
a) An example of how the horizontal bone is measured; b) An example of how the vertical bone is measured.

The Inverta design differs from the parallel-walled internal conical implant in two main aspects: Its body expands in the midsection to achieve maximum apical anchorage, creating a 2-3mm buccal gap without compressing the coronal bone; and it incorporates a 12° angled platform, allowing central placement within the extraction socket, optimizing residual alveolar bone use, and improving the prosthetic emergence profile for better aesthetic outcomes. Inclusion and Exclusion Criteria The inclusion criteria were as follows: American Society of Anesthesiologists (ASA) type I patients; age 18 years (16 , 17); good oral hygiene, with a plaque index &lt;15% according to the Löe Index (18); motivation and compliance with scheduled follow-up visits; presence of a single maxillary tooth (central or lateral incisors, canines, or premolars) indicated for extraction due to poor prognosis; absence of buccal bone dehiscence and presence of intact soft tissues without recession (Elian et al., type I alveolus (19)); adequate bone volume to achieve primary implant stability; and absence of adjacent bridges or amalgam restorations that could cause CBCT artifacts. Exclusion criteria included: Acute infection at the extraction site; missing posterior support that could lead to occlusal overload; insufficient mesio-distal space to accommodate a 4.5mm diameter implant (given that a 3.5mm coronal implant widens to 4.5mm at its middle third); unstable periodontal status; smoking more than 10 cigarettes per day; and systemic medical conditions contraindicating implant surgery. Patient Confidential Information, Informed Consent and Ethics Committee The study was carried out after receiving approval from the Clinical Research Ethics Committee (CREC) of the UIC; protocol number: CIR-ECL 2021-09, approved on 15 March 2022. It was designed in accordance with the ethical principles of the Declaration of Helsinki (Assembly 2013) (20). All patients received both verbal and written information about the treatment and the clinical study, including the advantages and disadvantages of participation. Two copies of the written informed consent approved by the CREC, together with an additional consent form for implant surgery from the University Dental Clinic (UDC), were provided. Once the patient had given consent, the surgery was scheduled. Randomization Patients were randomly assigned to either the co-axis or tapered implant group using a computer-generated sequence (www.randomization.org). Allocation was concealed with sealed opaque envelopes, which were opened by an independent investigator at the time of surgery to maintain blinding until tooth extraction. Surgical Procedure All surgical procedures were performed by six postgraduate students from the Master's Programme in Oral Surgery at the Universitat Internacional de Catalunya (UIC), introducing some operator variability among the 30 cases. Under local anesthesia (articaine 4% with 1:100,000 epinephrine), teeth were atraumatically extracted without flap elevation using elevators, forceps, and sectioning when required. The sockets were thoroughly debrided, irrigated with sterile saline, and the integrity of the buccal plate was verified with a periodontal probe. When the socket walls were intact, implant site preparation followed the manufacturer's drilling protocol using sequential burs under copious irrigation. Implant dimensions were selected to provide at least 5mm of apical anchorage and maintain a 2-3mm buccal gap. Due to its macrodesign, which narrows coronally to a 3.5-mm diameter neck and widens toward the middle third, the implant consistently created a residual buccal gap. This configuration represents an inherent advantage of the design, thereby facilitating graft placement without compromising primary stability. For optimal prosthetic emergence, co-axis implants were positioned centrally within the socket, while conventional conical implants were placed slightly palatally. A 1.5mm distance was maintained from adjacent roots, and implants were submerged approximately 4mm below the gingival margin. Buccal gaps 2mm were filled with deproteinized bovine bone mineral (Bio-Oss®, Geistlich). A subepithelial connective tissue graft was obtained from the tuberosity, or from the palate when necessary, using a split-thickness pocket incision and was stabilized with absorbable sutures. Prosthodontic Procedure Implant stability was evaluated by resonance frequency analysis (RFA) using the Penguin device (Integration Diagnostics, Sweden). When ISQ values exceeded 62, an immediate screw-retained provisional was placed; otherwise, a customized healing abutment or Maryland bridge was used to preserve peri-implant tissues. A standardized 3D-printed provisional crown (EXOCAD, Cerasmart) was left infra-occluded and adjusted after 12 weeks to optimize soft-tissue contours. Definitive restorations were delivered after three months using screw-retained E-max or zirconia crowns on titanium-base abutments, tightened at 35 Ncm and rechecked after two weeks. Cement-retained crowns were used only when screw access was buccally positioned. Variables Survival and Success of Implants Survival rate was determined as "the presence of the implant without extraction due to biological or mechanical complications". To evaluate implant success of the implants placed at 1 year of follow-up, the health scale for dental implants proposed by Misch et al. 2008 (21) was used. The classification has four categories. Success (optimum health) is defined by the absence of pain or tenderness during function, no implant mobility, less than 2mm of radiographic bone loss since surgery, and no history of exudates. Satisfactory survival also requires no functional pain and no mobility, but allows for 2-4mm of bone loss without exudates. Compromised survival is characterized by possible sensitivity on function, no mobility, bone loss greater than 4mm but involving less than half the implant body, probing depths exceeding 7mm, and a possible history of exudates. Finally, failure (clinical or absolute) is diagnosed when one or more of the following are present: Pain on function, implant mobility, bone loss exceeding half the implant length, uncontrolled exudates, or complete implant loss (21). Primary Stability of Implants Immediately after the surgery and before immediate loading, RFA measurements were recorded using the Penguin device (Integration Diagnostic Sweden AB, Göteborg, Sweden). Following the instructions, the SmartpegTM was screwed to each implant with 5Ncm of torque, and two measurements were made from two different angles (90 degrees at the buccal level and on the palatal aspect) and the highest value was recorded and the other discarded (22). Marginal Bone Loss Bone loss was assessed using CBCT. Although its use may raise concerns due to radiation exposure, current literature supports CBCT before surgery, after implant placement, and during follow-up, as modern technology allows minimal and reduced doses while optimizing image quality. In this study, scans were limited to the maxilla, further minimizing exposure. CBCT provided essential information for treatment planning, enabled postoperative verification of implant positioning, and confirmed the absence of complications after one year (23 , 24). a) CBCT analysis: To evaluate the marginal bone surrounding the implants, three CBCT scans were carried out using iCAT (Imaging Science International, LLC, Hatfield/USA), at three time points, and changes in volume were measured by overlaying the stereolithography files (STL): - CBCT 1: Prior to extraction for diagnosis and planning. This CBCT was performed by placing an individual bite fork from the LimaGuide system (Barcelona, Spain) in the patient's mouth. To ensure all CBCTs wew registered in the same craniomaxillary position, a bite registration material (Occlufast Rock, Zhermack) was placed on the fork, not exceeding 3mm thick, and used in the patient's mouth, the same method was used in the same fork in the other CBCTs. - CBCT 2: Immediately after implant placement - CBCT 3: After 1 year of follow-up, again always with the fork in the patient's mouth. b) CBCT superposition: First-year bone loss was assessed by overlapping CBCT 1, 2 and 3 using the LimaGuide software (Barcelona, Spain). The STL files from all three CBCTs were imported to measure volumetric changes. All the CBCTs were made with the individualized fork, which has four metal points, allowing accurate superimposition and providing reproducible measurements. c) Measurements of bone changes: Once the CBCTs were superimposed, the LimaGuide software was used, but this time the measurements was made in a 2D Digital Imaging and Communications in Medicine (DICOM) and 3 measurements were made in each aspect and the average was calculated. The following aspects were evaluated: - Horizontal measurement (Figure 2a): To determine if there were any volumetric changes in the horizontal aspect, two reference lines were made: A vertical line that divided the implant into two equal parts and a horizontal line that was perpendicular to this vertical line. With these two reference lines, 3 more horizontal lines parallel to the orange line, (1-3-5mm from the starting point), were used to obtain the horizontal bone changes. The distance of these lines were from the most buccal aspect to the palatal area. - Vertical measurement (Figure 2b): To determine the vertical bone changes, a reference line was made following the implant platform. With this reference line, two parallel lines were made: The most buccal bone peak; the most palatal bone peak. The distance between them determined the vertical bone changes. Soft Tissue Maintenance The tool that was used to assess the status of the soft tissues was the Pink Esthetic Score (PES) at one year of follow-up.

2

The PES is based on seven variables: Mesial papilla, distal papilla, soft tissue contour, gingival margin level, alveolar process deficiency, soft tissue color and texture. Each variable was evaluated with a score of 2-1-0, with 2 being the best score and 0 the worst score. Comparisons were made with the corresponding teeth (for example, if we were evaluating the 1.2 implant, it was compared with the natural tooth of 2.2). The maximum score, which reflects a perfect coincidence of the peri-implant soft tissue with that of the reference tooth, was 14 (25). Statistical Analysis Intra-examiner reliability was assessed using four variables: Buccal bone height, palatal bone height, ridge width at 1mm from the crest, and seven parameters of the Pink Esthetic Score (mesial papilla, distal papilla, gingival margin, gingival contour, alveolar process, color, and texture). The same measurements were repeated after one week by a blinded examiner, and the Intraclass Correlation Coefficient (ICC) was calculated to determine measurement consistency. Descriptive statistics included mean, standard deviation, minimum, maximum, median, and quartiles; categorical variables were summarized using absolute and relative frequencies. As several variables showed deviations from normality and the sample size was limited (n = 30), non-parametric methods were prioritized, with analyses focusing on medians and distributional differences. Inferential procedures comprised the Mann-Whitney U test for comparisons between two independent groups, the Wilcoxon signed-rank test for paired samples, and the Student's t-test when assumptions of normality and homoscedasticity were met. Associations between categorical variables were examined with the Chi-square test, while Spearman's correlation assessed monotonic relationships. For comparisons involving more than two groups, the Kruskal-Wallis test was applied, and longitudinal or factorial data were analyzed using the Brunner-Langer test. Statistical significance was set at p&lt;0.05. All analyses were performed with R 4.3.1 (R Core Team, 2023; Vienna, Austria. URL: http://www.R-project.org) and SPSS 15.0 (Chicago, IL, USA).

## Results

Baseline Characteristics and Homogeneity The sample included 30 patients (11 males, 36.7%; 19 females, 63.3%) with a mean age of 53.4±11.8 years (range: 30.8-78.8). Regarding tooth type, incisors represented 33.3% of cases (26.7% in the control group, 40.0% in the test group), canines 16.7% (13.3% vs. 20.0%), and premolars 50.0% (60.0% vs. 40.0%). Implant diameters of 3.5mm accounted for 50.0% of placements (46.7% vs. 53.3%), 4.0mm for 46.7% (53.3% vs. 40.0%), and 4.5mm for 3.3%, exclusively in the test group (6.7%). The most frequent implant length was 13.0mm, slightly more common in the test group; shorter implants (11.0mm) predominated in the control group, while 11.5mm implants were more frequent in the test group. Baseline comparability between groups was confirmed using Chi-square and independent t-tests, showing no significant differences in gender (p=0.130), age (p=0.790), tooth type (p=0.549), implant diameter (p=1.000), or implant length (p=0.441). The intra-examiner reliability analysis demonstrated excellent measurement consistency, with an ICC of 0.91 (95% CI, 0.86-0.95) for the repeated CBCT and PES measurements. Bone dimensions were measured at baseline (T0), immediately after extraction and implant placement (T1), and after one year (T2), with changes assessed between T1-T0, T2-T1 and T2-T0. Implant Survival and Success Implant survival was 100%, as no implant was lost due to biological or mechanical complications. Implant success was defined by osseointegration and functionality, distinguishing between absolute success and satisfactory survival; no cases of compromised success or failure were observed. The overall success rate was 83.3%. In the control group, 93.3% of implants (n=14) met the success criteria-absence of pain, lack of mobility, &lt;2mm radiographic bone loss, and no exudates-while 6.7% (n=1) were classified as satisfactory survival, showing 2-4mm of bone loss but maintaining clinical stability. In the test group, 73.3% (n=11) achieved success, and 26.7% (n=4) were categorized as satisfactory survival. At the 1-year follow-up, no significant differences were observed between groups (p=0.327). Implant Stability Insertion Torque (IT): Primary stability was assessed by IT, recorded as the maximum torque value (Ncm) displayed by the surgical motor during implant placement, reflecting the mechanical engagement between implant threads and surrounding bone. In the control group, the median IT was 35.0 Ncm (95% Confidence Interval [CI]: 26.17-37.16; SD=9.9), with values ranging from 11 to 45 (Q1=25, Q3=40). In the test group, the median IT was 42.0 Ncm (95% CI: 32.91-49.23; SD=14.7), ranging from 16 to 60 (Q1=27, Q3=55). Although this difference was not statistically significant (p=0.074), the trend suggested that Inverta implants may achieve higher initial stability. Implant Stability Quotient (ISQ): Resonance frequency analysis (RFA) was performed in buccal (90°) and palatal directions. In the control group, buccal ISQ had a mean of 56 (95% CI: 46.8-64.6; SD=16.1), range 23-78, and a median of 56.0 (Interquartile Range [IQR]: 50.0-71.0). Palatal ISQ presented a mean of 60 (95% CI: 50.2-67.5; SD=15.7), range 23-78, and a median of 60.0 (IQR: 51.0-71.0). In the test group, buccal ISQ values were higher, with a mean of 62 (95% CI: 58.9-64.8; SD=5.3), range 51-72, and a median of 62.0 (IQR:60.0-64.0). Palatal ISQ was comparable, with a mean of 62 (95% CI: 61.1-64.4; SD=3.0), range 58-67 and a median of 62.0 (IQR: 60.0-66.0). Although slightly higher ISQ medians were observed in the test group for both directions, inter-group differences were not statistically significant. Provisionalisation: The choice of provisional restoration was determined by implant stability. In the control group, 20% of cases received an immediate aesthetic provisional, while 80% were restored with a customised healing abutment due to lower stability. In the test group, 40% received an immediate aesthetic provisional and 60% a customized abutment, as most implants presented ISQ values &lt;62. Chi-square analysis (p=0.426) revealed no significant differences between groups regarding the type of provisionalization. Horizontal Bone Loss Horizontal bone changes were evaluated at 1mm, 3mm and 5mm between baseline (T0) and 1 year (T2) (Table 1).

Table 1

At 1mm, the global median reduction was -0.83mm. In the control group, the median was -0.19mm (mean -0.55mm; 95% CI: -1.38 to 0.27; SD 1.49), whereas in the test group it was -0.97mm (mean -1.65mm; 95% CI: -1.85 to -0.43; SD 2.18). Bone loss at this level was significant over time (p&lt;0.001) and followed a similar pattern in both groups (p=0.379), with no significant differences between them (p=0.208). At 3mm, the global median was -0.45mm. The control group showed a median of -0.08mm (mean 0.22mm; 95% CI: -0.42 to 0.87; SD 1.18), while the test group presented -0.53mm (mean -0.69mm; 95% CI: -1.27 to -0.10; SD 1.05). Bone reduction at this level was also significant over time (p=0.035), occurring similarly in both groups (p=0.133), with no significant differences between them (p=0.400). This conclusion was consistent across all time points (p=0.133). At 5mm, the global median change was -0.25mm. In the control group, the median was -0.29mm (mean 0.01mm; 95% CI: -0.65 to 0.68; SD 1.21), and in the test group -0.20mm (mean -0.31mm; 95% CI: -0.92 to 0.31; SD 1.13). The reduction at this level showed marginal significance over time (p=0.070) and was similar between groups (p=0.253). No significant differences were detected between groups (p=0.492), consistent across different evaluation times (p=0.253). Vertical Bone Loss Vertical bone loss was assessed in both the buccal and palatal regions between T1 (implant placement) and T2 (1-year follow-up) (Table 2).

Table 2

At the buccal region, the global median reduction was -0.58mm. In the control group, the median loss was -0.38mm (mean -0.57mm; 95% CI: -0.92 to -0.22; SD 0.63), with values ranging from -1.94 to 0mm (Q1= -1.04; Q3=0). In the test group, the median was -0.86mm (mean -0.92mm; 95% CI: -1.33 to -0.51; SD 0.73), ranging from -0.59 to 0.03mm (Q1= -1.15; Q3= -0.42). These results indicate a greater median loss in the test group compared to the control group, although both showed relatively contained variability, as reflected by the interquartile ranges. At the palatal region, the global median loss was -0.59mm. The control group exhibited a median of -0.74mm (mean -1.05mm; 95% CI: -1.64 to -0.46; SD 1.06), with values between -0.16 and 0mm (Q1= -1.74; Q3=0). In the test group, the median was lower at -0.34mm (mean -0.69mm; 95% CI: -1.12 to -0.25; SD 0.78), ranging from -2.48 to -0.04mm (Q1= -1.15; Q3= -0.12). These data suggest that, unlike the buccal aspect, the test group tended to preserve slightly more palatal bone height compared to the control group, although dispersion was broader due to the presence of more extreme values. Taken together, vertical bone loss was modest in both regions and groups, with differences that suggest site-specific variation rather than consistent group-dependent effects. Buccal bone reduction appeared slightly greater in the test group, whereas palatal loss was more pronounced in the control group. Overall Horizontal and Vertical Changes Table 3 summarizes the global horizontal and vertical bone changes, together with their statistical significance when comparing control and test groups.

Table 3

No significant intergroup differences were detected at any level. For horizontal changes, at 1mm the Brunner-Langer test showed no significant differences between groups (p=0.208), and the overall comparison from T0 to T2 also lacked significance (p=0.178). At 3mm, intergroup differences were similarly non-significant (p=0.400), and changes over time from T0 to T2 showed only a trend without reaching significance (p=0.089). At 5mm, neither the between-group comparison (p=0.492) nor the longitudinal assessment (T0-T2: p=0.648) demonstrated statistical significance. For vertical changes, buccal bone height reduction did not differ significantly between groups (p=0.124), and the same was observed for the palatal aspect (p=0.533). Taken together, these analyses indicate that although some trends were observed, particularly at the 3mm horizontal level, neither horizontal nor vertical bone changes differed significantly between the test and control groups throughout the study period. Aesthetic Evaluation (PES) The aesthetic outcome of implant restorations was assessed using the PES, a soft tissue-oriented index ranging from 0 to 14, with higher values reflecting better peri-implant esthetics. At the 1-year follow-up, the control group presented a median PES of 12 (IQR 10-12), with a mean of 11.4 (95% CI: 10.38-12.42), SD 1.8, and values ranging from 7 to 14. The test group showed a median PES of 11 (IQR 9-13), with a mean of 11.2 (95% CI: 10.13-12.27), SD 1.9, and scores between 9 and 14. These results indicate very similar central tendencies between groups, with only a 1-point difference in medians and substantial overlap of the confidence intervals, consistent with comparable aesthetic performance at one year. Dispersion was modest in both groups, although the test group displayed a slightly wider IQR, suggesting greater variability but a similar overall aesthetic level. Inferential analysis revealed no significant associations between PES and any of the other evaluated variables (all p&gt;0.05), indicating that aesthetic outcomes were relatively independent of the mechanical and dimensional parameters of the implants in this study.

## Discussion

This randomized clinical trial evaluated 30 patients undergoing immediate post-extraction implant placement in the anterior maxilla, with homogeneous baseline characteristics between groups. At the 1-year follow-up, implant survival was 100% in both groups, with no biological or mechanical failures. The overall success rate was 83.3%, with higher values in the control group (93.3%) compared to the test group (73.3%). According to the Misch Classification, the distinction between "success" and "satisfactory survival" is determined by the degree of radiographic bone loss: &lt;2mm is defined as success, whereas 2-4mm corresponds to satisfactory survival. Studies using the body-shift (Inverted Co-Axis) design have reported success rates of 93.3% (Vandeweghe et al. (11)) and 92.95% after 5 years (Ma et al. (14)), while Brown et al. (10) reported 91.6% for conventional conical implants under immediate loading. The lower success observed in our test group may be partly due to the use of CBCT for volumetric analysis, which can overestimate marginal bone loss compared with periapical radiographs-shown to be less precise by the ICOI 2008 consensus (21). Additionally, the fact that all surgeries were performed by postgraduate students may have introduced operator-related variability compared to studies involving experienced clinicians. The 100% survival rate in our series is consistent with Östman et al. (26), who reported 98.7% survival at 18-24 months with conventional conical implants, and Chu et al. (27), who observed 100% survival at one year using tapered implants. Galve-Huertas et al. (28) also reported 100% survival at one year, but only in inverted body-shift implants. Across several studies (26 - 30), survival consistently exceeds 95%, though reported success rates vary due to differences in definitions and outcome criteria. For example, Östman et al. (26) did not provide a specific success percentage but noted stable hard/soft tissues and high aesthetic satisfaction, whereas Levin et al. (29) emphasized reliable osseointegration and tissue preservation without quantifying success. These discrepancies underscore the need for standardized outcome reporting when assessing innovative implant designs. Primary stability remains essential for the success of immediate loading protocols (7 - 9). In this study, insertion torque (IT) was slightly higher in the test group (median 42.0 Ncm; IQR 27-55) compared with the control group (35.0 Ncm; IQR 25-40), although the difference was not statistically significant (p=0.074). Both implant types achieved torque values within the range considered optimal for immediate loading. Similar IT levels have been reported in the literature for comparable designs: Brown et al. (10) recorded mean values of 38.6±8.1 Ncm for conventional conical implants, Ma et al. (14) reported 41.7±10.2 Ncm for tapered implants, and Galve-Huertas et al. (28) found 40.5±15.7 Ncm for inverted body-shift implants. These results collectively confirm that the inverted configuration does not compromise primary mechanical stability. Resonance frequency analysis (RFA) further supported these findings. The test group exhibited slightly higher ISQ medians both buccally (62.0; IQR 60-64) and palatally (62.0; IQR 60-66) than the control group (56.0; IQR 50-71 and 60.0; IQR 51-71, respectively), though without statistical significance. Comparable stability trends have been reported by Chu et al. (30), who observed mean ISQ values of 65.3±5.7 regardless of implant geometry, and by Brown et al. (10), showing an increase from 64.7±5.2 at placement to 69.5±6.1 after one year in conical implants. Similarly, Ma et al. (14) reported a rise from 65.1±4.38 to 69.9±4.94 over five years with tapered implants, while Galve-Huertas et al. (28) documented ISQ values of 62.6±3.1 (0°) and 61.6±9.7 (90°) for inverted implants, with a moderate correlation to IT (r=0.52). Altogether, these results emphasize that both torque and multidirectional ISQ are reliable indicators of implant stability and can effectively guide immediate provisionalization in anterior maxillary cases. Horizontal bone remodeling followed the expected physiological pattern, with most resorption occurring coronally. The mean horizontal reduction was -0.83mm at 1mm, significant over time (p&lt;0.001), but with no differences between implant designs (p=0.208). At 3mm (-0.45mm) and 5mm (-0.25mm), dimensional changes were smaller and not statistically significant. Similar results have been reported in previous studies: Galve-Huertas et al. (28) observed a -0.65mm loss at 1mm with inverted implants (p=0.015), while Chu et al. (27) and Chu et al. (30) found minimal labial plate changes at one year (0.07-0.34mm) with tapered designs. Östman et al. (26), analyzing conical implants, also reported stable values at 1mm (1.82mm) over 18-24 months, with only minor variation at deeper levels. Collectively, these findings confirm that horizontal remodeling is concentrated in the coronal third, whereas apical levels remain largely stable, irrespective of implant macro-design. Vertical remodeling exhibited site-specific behavior. Buccal resorption was greater in the test group (-0.86mm) than in controls (-0.38mm), while palatal reduction was more pronounced in controls (-0.74mm) compared with the test group (-0.34mm). Comparable patterns were described by Galve-Huertas et al. (28), who found buccal and palatal height losses of -0.65mm (p=0.003) and -0.42mm (p=0.002), respectively. These results suggest that vertical changes are mainly influenced by biological remodeling and surgical factors rather than implant geometry, as also indicated by Levin et al. (29) and Östman et al. (26). However, the slightly greater buccal remodeling in the test group could be explained by the smaller buccal gap inherent to its central positioning within the socket, which reduces the space for graft material and may accentuate remodeling of the thin cortical plate. From a clinical perspective, the magnitudes of horizontal and vertical bone loss observed in both implant designs fall within the range typically considered compatible with long-term peri-implant stability and aesthetic preservation. Marginal bone changes below 1mm after one year are regarded as clinically acceptable and unlikely to compromise soft-tissue support or prosthetic emergence. Thus, although the differences between groups were not statistically significant, the observed remodeling should be interpreted as part of a normal physiological response rather than an effect of implant design. Aesthetic outcomes, assessed with the PES, were similar between groups, with medians of 12 (control) and 11 (test), and no significant differences. These results are in line with Galve-Huertas et al. (28), who reported a median PES of 11.5 (IQR 10-12.5) for inverted implants at one year, Östman et al. (26), who described mean values of 12.63 at 6 months and 13.0 at 24 months in conical implants, and Chu et al. (27), who found a mean PES of 12.5 at one year in tapered implants. Levin et al. (30) emphasized the importance of soft-tissue integration, while Chu et al. (30) confirmed that implant design can support favorable contours when combined with appropriate surgical protocols. Collectively, these findings suggest that aesthetic outcomes depend more on soft-tissue management than on implant macro-design. This study has several limitations. The small sample size limits the statistical power, and the one-year follow-up does not allow conclusions to be drawn regarding long-term stability. Surgeries were performed by six different postgraduate operators, which may have introduced variability in surgical technique. In addition, the inclusion of different tooth types-from central incisors to premolars-could have influenced the clinical and radiographic outcomes due to anatomical differences in socket morphology and bone thickness. Variations in implant positioning also complicated standardized bone measurements, and digital superimposition presented technical challenges. Moreover, the multivariable analysis did not reveal significant associations between the investigated variables. Future research should involve larger cohorts, longer follow-up periods, standardized placement protocols, and advanced digital technologies to improve measurement accuracy and facilitate comparison across studies.

## Conclusions

Both the novel inverted body-shift (co-axis 12°) and the conventional conical implants showed comparable clinical outcomes for immediate post-extraction placement in the anterior maxilla. Implant survival was 100% in both groups, with an overall success rate of 83.3% and no significant differences, despite a higher absolute success in the control group (93.3% vs. 73.3%). Primary stability, assessed by insertion torque and ISQ, was adequate for immediate loading in all cases. Marginal bone remodeling was limited at all levels, with no significant intergroup differences in horizontal or vertical changes. Slight buccal resorption in the test group and palatal loss in the control group likely reflected anatomical variation rather than implant design. Aesthetic outcomes (PES) were similarly high and stable across groups, confirming the clinical viability of both systems for immediate placement in the aesthetic zone. The inverted co-axis design, however, may offer practical advantages in challenging cases. Its body-shift geometry favors central alveolar positioning, improved stability, and screw-retained restorations with optimal emergence profiles. These results should be interpreted within the study's limitations-small sample size, one-year follow-up, and operator variability. Larger, long-term studies are warranted to confirm these findings.

## Figures and Tables

**Table 1:** Table Horizontal bone loss changes between baseline (T0) and follow-up (T2) at different levels (1, 3 and 5mm).

		Mean (CI 95%)	SD	Min	Max	Q1	Median	Q3
Control Group	Horizontal changes at 1mm T2-T0	-0.55 (-1.38-0.27)	1.49	-3.19	1.57	-2.05	-0.19	0.47
	Horizontal changes at 3mm T2-T0	0.22 (-0.42-0.87)	1.18	-1.66	1.97	-0.83	-0.08	1.46
	Horizontal changes at 5mm T2-T0	0.01 (-0.65-0.68)	1.21	-1.58	2.22	-0.89	-0.29	0.97
Test Group	Horizontal changes at 1mm T2-T0	-1.65 (-1.85-0.43)	2.18	-7.83	0.62	-1.9	-0.97	-0.42
	Horizontal changes at 3mm T2-T0	-0.69 (-1.27- -0.10)	1.05	-3.28	0.82	-0.98	-0.53	-0.18
	Horizontal changes at 5mm T2-T0	-0.31 (-0.92-0.31)	1.13	-2.43	1.6	-0.9	-0.2	0.67

CI: Confidence interval. SD: Standard deviation. Q1: Percentile 25. Q3: Percentile 75.

**Table 2:** Table Vertical bone loss changes (T2–T1) in buccal and palatal bone height for control and test groups.

		Mean (CI 95%)	SD	Min	Max	Q1	Median	Q3
Control Group	Buccal height at T2-T1	-0.57 (-0.92- -0.22)	0.63	-1.94	0	-1.04	-0.38	0
	Palatal height at T2-T1	-1.05 (-1.64- -0.46)	1.06	-0.16	0	-1.74	-0.74	0
Test Group	Buccal height at T2-T1	-0.92 (-1.33- -0.51)	0.73	-0.59	0.03	-1.15	-0.86	-0.42
	Palatal height at T2-T1	-0.69 (-1.12- -0.25)	0.78	-2.48	-0.04	-1.15	-0.34	-0.12

Vertical bone loss changes (T2–T1) in buccal and palatal bone height for control and test groups.

**Table 3:** Table Horizontal and vertical bone changes and corresponding statistical significance.

	p-value (Brunner-Langer)	p-value T2-T0	p-value T2-T1
H 1mm	0.208	0.178	--
H 3mm	0.4	0.089	--
H 5mm	0.492	0.648	--
V buccal	--	--	0.124
V palatal	--	--	0.533

*p<0.05. **p<0.01. ***p<0.001. “--” indicates non-applicable comparisons (not performed for that variable).

## Data Availability

Declared none.
